# Teamwork-Oriented Physical Education Course Organisation and Survey-Wave Differences in Physical Activity, Sitting Time, and Perceived Physical Literacy Among Medical Students: A Two-Wave Unlinked Parallel-Group Evaluation

**DOI:** 10.3390/bs16101948

**Published:** 2026-10-20

**Authors:** Guochun Liu, Ke Jiang, Jingrong Wang, Wen Li, Jiaqi Ling, Hui An, Xiaoxiao Huang

**Affiliations:** 1 College of Exercise Medicine, Chongqing Medical University, Chongqing 400016, China; lgc890206@163.com (G.L.); 100676@cqmu.edu.cn (K.J.); 102553@cqmu.edu.cn (H.A.); 2 Division of Sports Science and Physical Education, Tsinghua University, Beijing 100084, China; 3 Faculty of Education, The Chinese University of Hong Kong, Sha Tin, New Territories, Hong Kong 999077, China; 1155254832@link.cuhk.edu.hk; 4 School of Public Health, Chongqing Medical University, Chongqing 400016, China; 2025223128@stu.cqmu.edu.cn (W.L.); 2025223129@stu.cqmu.edu.cn (J.L.)

**Keywords:** medical students, physical education, physical activity, sitting time, perceived physical literacy, teamwork-oriented instruction, non-randomised evaluation

## Abstract

Medical students face health risks associated with high academic demands and limited discretionary time, yet evidence remains insufficient on whether health-promoting strategies embedded within existing curricula can improve their health-related behaviours and outcomes. We conducted a non-randomised, two-wave unlinked parallel comparison within a compulsory physical education course. Across four survey cells, 354 questionnaire responses, rather than 354 unique longitudinal participants, were analysed. Physical activity and sitting time were assessed using the International Physical Activity Questionnaire–Short Form, perceived physical literacy using the Perceived Physical Literacy Instrument, academic burnout using the Maslach Burnout Inventory–Student Survey, psychological distress using the Depression Anxiety Stress Scales-21, and sleep quality using the Pittsburgh Sleep Quality Index. Differential changes were estimated using Group × Wave models. Relative to the comparison condition, the teamwork-oriented condition showed more favourable changes in moderate-to-vigorous physical activity (MVPA) (67.15 min/week, 95% CI 2.23 to 132.07; *p* = 0.043) and sitting time (−87.08 min/day, 95% CI −159.28 to −14.88; *p* = 0.018); BH-FDR-adjusted *q* values were 0.058 and 0.054. Perceived physical literacy showed a favourable but imprecise estimate (2.11 points, 95% CI −0.07 to 4.29; *q* = 0.058). No clear differential changes were detected for academic burnout, psychological distress, or sleep quality. The focal outcomes showed favourable estimated patterns, but none met the conventional 0.05 threshold after multiplicity adjustment. Given the non-randomised design, unlinked surveys, and complete confounding of instructor with course condition, the findings should be interpreted as differences between two specific instructor–course contexts rather than as causal effects of the instructional approach.

## 1. Introduction

Medical education shapes both future professional practice and students’ own health behaviours. Intensive coursework, sustained academic demands, and prolonged sitting make physical activity particularly susceptible to being displaced from medical students’ daily routines, while insufficient physical activity has consistently been associated with burnout and poorer quality of life among medical students (Morgan et al., 2024; Taylor et al., 2022). This issue extends beyond individual student health because future physicians will also influence patients’ physical activity promotion and lifestyle management (Borges et al., 2024). Meanwhile, education on exercise counselling and exercise prescription remains relatively limited within medical curricula, and existing curricular interventions have primarily improved students’ knowledge, confidence, and willingness to provide counselling, whereas evidence remains insufficient as to whether such interventions translate into sustained changes in students’ own physical activity behaviours (Shivgulam et al., 2024). Accordingly, medical students’ physical activity is relevant not only to student health but also to the broader educational context in which future health-promoting practice is developed.

However, evaluating a physical education curriculum solely in terms of increased physical activity may overlook other relevant behavioural outcomes. Physical activity and sedentary behaviour are related but are not simple opposites; a student may engage in substantial amounts of moderate-to-vigorous physical activity while still accumulating prolonged sitting time because of academic demands. Intervention studies among university students have likewise shown that increases in physical activity do not necessarily coincide with concurrent reductions in sedentary behaviour (Brown et al., 2024; Castro et al., 2020). Physical literacy provides a complementary framework for considering the physical competence, motivation and confidence, and interaction with the environment that may support continued participation in physical activity. Physical literacy is an important behavioural construct linking the ability and willingness to participate with actual engagement in physical activity (Cairney et al., 2019). Meta-analytic evidence supports a positive association between physical literacy and physical activity, although the strength of this relationship varies across measurement and activity characteristics (Ying et al., 2025). Studies among Chinese medical students have also identified associations among physical literacy, physical activity, and health-related outcomes (X. Yang et al., 2024). These findings raise the question of whether different approaches to organising physical education are associated with students’ whole-week physical activity and sitting patterns and with perceived physical literacy. Whether any such differences extend specifically beyond scheduled lessons or persist after the course requires separate investigation. Existing evidence leaves a specific gap at the intersection of medical education and physical education. Curricular studies involving medical students have largely focused on exercise-counselling competence, physical fitness, or health-related knowledge, whereas research on cooperative or game-enhanced physical education has primarily examined engagement and educational outcomes in broader student populations (Boke et al., 2025; Paez et al., 2024; M. Yang et al., 2026). Consequently, little is known about whether teamwork-oriented organisation within an existing physical education curriculum is associated with concurrent differences in physical activity, sitting time, and perceived physical literacy among medical students. Burnout, psychological distress, and sleep are related to physical activity, but they are also jointly influenced by multiple factors, including the academic demands of medical training, assessment-related pressure, and semester-related rhythms. In the present study, these outcomes were therefore examined exploratorily as broader health-related outcomes rather than as direct indicators of the effectiveness of the course organisation.

Accordingly, we evaluated two instructional-organisation contexts embedded within the same compulsory physical education curriculum (Des Jarlais et al., 2004; Hoffmann et al., 2014; von Elm et al., 2007). The primary objective was to compare survey-wave differences in self-reported moderate-to-vigorous physical activity, sitting time, and perceived physical literacy between the two contexts. Academic burnout, psychological distress, and sleep quality were examined as exploratory outcomes. Because activity during scheduled lessons could not be separated from activity elsewhere and no post-course follow-up was conducted, the study did not evaluate out-of-class transfer or maintenance after course completion.

## 2. Materials and Methods

### 2.1. Study Design and Setting

This single-centre, non-randomised, two-wave unlinked parallel-group course evaluation was conducted among first-year undergraduate medical students enrolled in public physical education at Chongqing Medical University. Reporting was informed by STROBE and TREND, and the course description by TIDieR (Des Jarlais et al., 2004; Hoffmann et al., 2014; von Elm et al., 2007). A completed TIDieR checklist is provided in Supplementary File S1. Course conditions were determined by existing class arrangements rather than individual randomisation, with one instructor assigned to each condition. The spring semester began on 2 March 2026, with 16 scheduled teaching weeks (2 March–21 June) followed by two assessment weeks (22 June–5 July). Both conditions completed baseline surveys on 4 March (week 1). Final surveys preceded the respective course examinations on 17 June (week 16) in the teamwork-oriented condition and 24 June (week 17) in the comparison condition. Thus, “end-of-course” referred to condition-specific pre-examination assessments rather than a common post-instruction time point. Actual teaching-session counts before the final surveys were unavailable (Supplementary File S1). Both survey waves were administered using Wenjuanxing (Changsha Ranxing Information Technology Co., Ltd., Changsha, China; https://www.wjx.cn/), a web-based questionnaire platform. Anonymous questionnaires lacked stable identifiers, precluding individual linkage across waves. Analyses therefore compared survey-cell means across waves rather than within-student changes.

### 2.2. Participants, Informed Consent, and Ethics

Eligible participants were first-year undergraduate medical students enrolled in the relevant physical education classes. The teamwork-oriented and comparison conditions each comprised three classes, with 95 and 94 enrolled students, respectively. At baseline, 94/95 (98.9%) students in the teamwork-oriented condition and 93/94 (98.9%) in the comparison condition completed the survey; at end-of-course, the corresponding figures were 93/95 (97.9%) and 76/94 (80.9%). One teamwork-oriented end-of-course response and one comparison baseline response did not meet the consent criterion and were excluded, yielding analytic cells of 94, 92, 92, and 76 responses, respectively (354 responses in total). Because surveys were unlinked, end-of-course respondents could not be identified as the same individuals who completed the baseline survey. The study was approved by the Medical Research Ethics Committee of Chongqing Medical University (2025-044; 30 July 2025) and conducted in accordance with the Declaration of Helsinki.

### 2.3. Course Condition and Parallel Comparison Condition

Both groups followed the university’s standardised 18-week Health and Physical Fitness Promotion course, comprising 16 scheduled teaching weeks and two assessment weeks. Each session during the teaching period lasted 80 min. Course content included sports injury prevention and first aid, long-distance running, jumping, strength training, sprinting, physical fitness test practice, Tai Chi, and swimming. The planned course duration, number of teaching weeks, session length, core content, learning objectives, schedule framework, and assessment requirements were the same across conditions. The principal contrast between conditions was the organisation of instruction during the 16-week teaching period.

**Figure 1:**
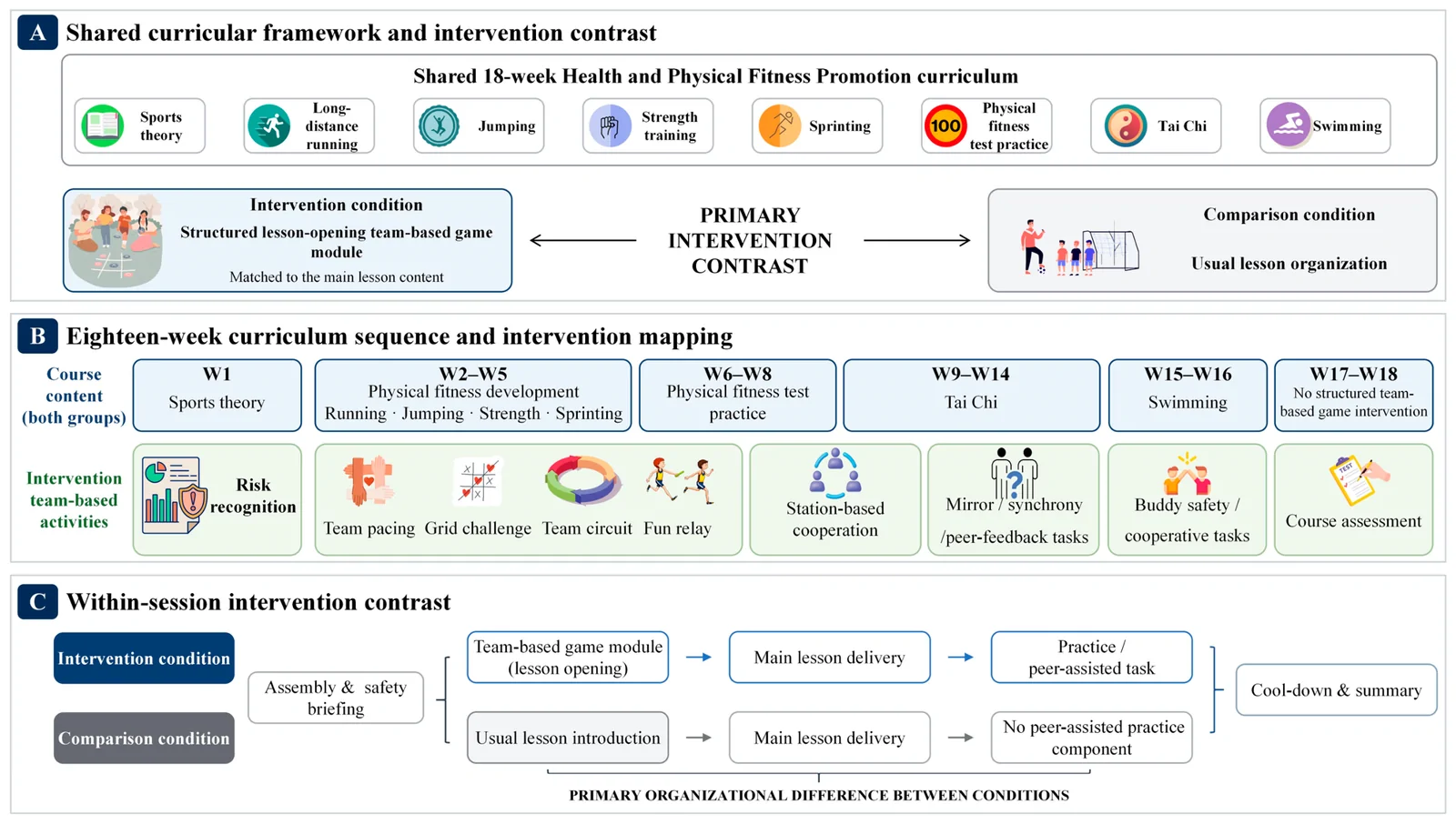
Course framework and organisational contrast between study conditions.

The teamwork-oriented condition incorporated a prescribed 25–30 min team-game block into each 80 min lesson, followed by small-group instruction and peer-assisted practice. The 6–12 min durations reported for individual tasks in Supplementary File S1 did not represent the full game block. The comparison condition followed the same curriculum without systematic team games or structured teamwork. Realised game duration, attendance, session-level fidelity, and in-class physical activity were not independently quantified for analysis. Extracurricular exercise time was recorded through routine check-ins but was not linked to the anonymous surveys. Intervention procedures are detailed in Supplementary File S1 and summarised in Figure 1.

### 2.4. Measurement Instruments and Outcome Definitions

Physical activity and sitting time were assessed using the 7-day recall version of the International Physical Activity Questionnaire–Short Form (IPAQ-SF) (Craig et al., 2003). The IPAQ-SF has undergone reliability and validity testing in multinational samples, and the Chinese 7-day short form has demonstrated acceptable test–retest reliability (Macfarlane et al., 2007). Activity bouts lasting <10 min were not counted, and values >180 min/day for a given intensity were truncated to 180 min/day. Vigorous activity, moderate-intensity activity, and walking were assigned 8.0, 4.0, and 3.3 METs, respectively. MVPA was defined as the weekly cumulative minutes of vigorous and moderate-intensity physical activity, sitting time was reported in min/day, and total physical activity was expressed as MET-min/week. Detailed data-cleaning and missing-data rules are provided in Table S3.

Perceived physical literacy was assessed using the 8-item Simplified Chinese version of the Perceived Physical Literacy Instrument (PPLI) (Sum et al., 2016). Structural validity of this version has been supported among undergraduate students in mainland China (Ma et al., 2020). Each item was rated on a 5-point Likert scale, yielding a total score of 8–40, with higher scores indicating higher perceived physical literacy. The PPLI total score was included in the focal outcome family, whereas the three subscales physical competence and confidence, motivation, and interaction with the environment were included in the secondary outcome family.

Academic burnout was assessed using the 15-item Maslach Burnout Inventory–Student Survey (MBI-SS), which includes emotional exhaustion, cynicism, and academic efficacy; each item was scored from 0 to 6 (Hu & Schaufeli, 2009; Schaufeli et al., 2002). Mean item scores were calculated separately for the three dimensions, and no overall burnout score was calculated; higher emotional exhaustion and cynicism indicated greater burnout, whereas academic efficacy was interpreted in the opposite direction.

Psychological distress was assessed using the 21-item Depression Anxiety Stress Scales (DASS-21). Each item was scored from 0 to 3, and the three 7-item subscales for depression, anxiety, and stress, as well as the 21-item total score, were calculated. Raw scores were retained; scores were not multiplied by 2, and no clinical severity categories were assigned (Lovibond & Lovibond, 1995; Lu et al., 2018).

Sleep quality was assessed using the Pittsburgh Sleep Quality Index (PSQI), which comprises seven components and yields a total score ranging from 0 to 21, with higher scores indicating poorer sleep quality. When the sleep-duration or sleep-efficiency component could not be reconstructed unambiguously, only the PSQI total score was set to missing; other valid outcomes from the same questionnaire were retained (Buysse et al., 1989; Zheng et al., 2016). The Chinese version has demonstrated reliability and validity among Chinese medical students, although research in Chinese undergraduate students suggests that its structure may not be strictly unidimensional (Guo et al., 2016).

### 2.5. Internal Consistency Analyses in the Present Sample

Internal consistency was estimated within each Group × Wave survey cell. Cronbach’s α and McDonald’s ω were calculated for the PPLI, MBI-SS, and DASS-21; the Spearman–Brown coefficient was used for the two-item PPLI interaction-with-the-environment subscale. PSQI α was calculated only for questionnaires in which all seven components could be reconstructed. Internal consistency was not evaluated for the IPAQ-SF because it comprises distinct behavioural domains. Cell-specific estimates are reported in Supplementary Table S4.

### 2.6. Statistical Analysis

Continuous variables were summarised as means and standard deviations; medians and interquartile ranges were additionally reported for skewed physical activity variables (Table S5). Categorical variables were summarised as frequencies and percentages. Baseline differences between conditions were described using standardised mean differences (SMDs), without significance testing for baseline comparisons. The adjustment set included sex, age, and college; year of study was not included because all participants were first-year students.

The main analyses used ordinary least-squares regression with HC3 heteroskedasticity-robust standard errors: Outcome = Group + Wave + Group × Wave + Sex + Age + College. The comparison condition and baseline served as reference categories. The interaction term estimated between-condition differences in survey-wave changes in mean outcomes. Because responses were unlinked, within-person correlation across waves could not be estimated. The reported HC3 confidence intervals and *p*-values were conditional on working independence at the survey-response level and did not account for overlapping respondents or shared-class dependence. Class-clustered inference was not performed given the six classes. Instructor and course-condition effects could not be separated because each condition had one instructor.

MVPA, sitting time, and the PPLI total score were treated as focal outcomes. Because no confirmatory hierarchy among these three focal outcomes had been prospectively specified, they were analysed as a single multiple-comparison family, with the Benjamini–Hochberg procedure used to control the false discovery rate (FDR). The three PPLI subscales, together with total physical activity, vigorous-intensity activity, moderate-intensity activity, and walking, formed the secondary outcome family. The three MBI-SS dimensions, three DASS-21 dimensions, and PSQI total score formed the exploratory psychological and sleep outcome family. BH-FDR correction was applied separately within each family. Effect estimates are reported with 95% confidence intervals, nominal two-sided *p* values, and BH-FDR-adjusted *q* values. Standardised interaction effects were calculated using the pooled baseline standard deviation of the two conditions. Post hoc observed power was not calculated; precision was evaluated using effect estimates and 95% confidence intervals. Sensitivity analyses included an unadjusted model, a model adjusted for sex and age, the fully adjusted model, median regression for MVPA, and log(1 + x) transformations of MVPA and sitting time. Statistical analyses were performed in Python 3.13.5 using pandas 2.2.3, NumPy 2.3.5, SciPy 1.17.0, statsmodels 0.14.6, and scikit-learn 1.8.0.

## 3. Results

### 3.1. Analytic Sample and Baseline Composition Between Groups

Because surveys were anonymous and unlinked, the lower end-of-course response rate in the comparison condition reflects differential wave participation rather than observed individual attrition. Owing to outcome-specific analytic availability, the effective sample sizes for MVPA, sitting time, and PSQI were 351, 343, and 351, respectively; the PPLI total score had no unavailable outcome values (*n* = 354). At baseline, standardised differences between groups were small for age, sex, MVPA, and sitting time (|SMD| ≤ 0.111). Larger standardised differences were observed for some college categories (maximum |SMD| = 0.225), PPLI total score (|SMD| = 0.279), and DASS-21 total score (|SMD| = 0.260) (Table 1).

### 3.2. Differential Changes in Physical Activity and Sitting Time

**Figure 2:**
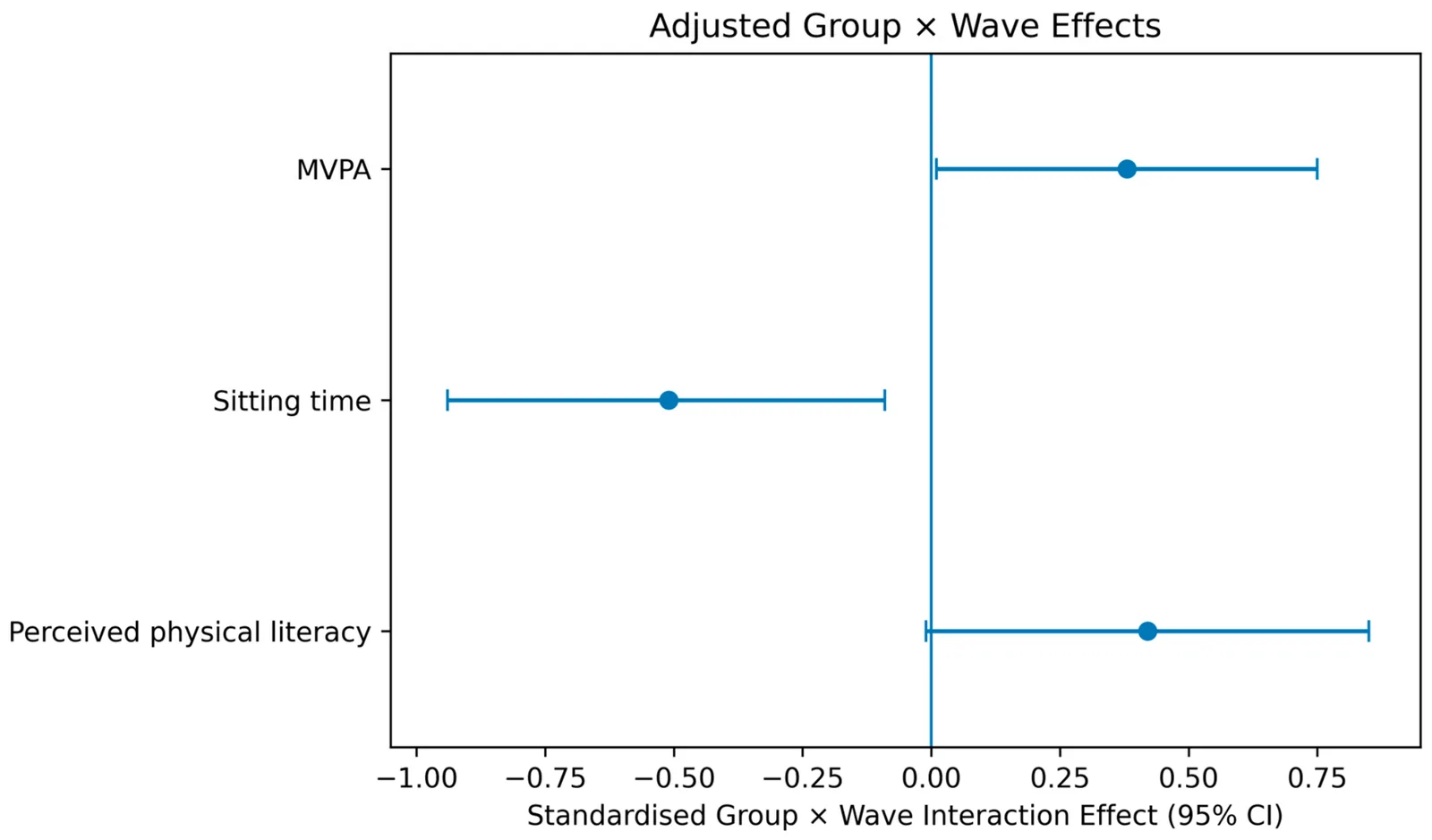
Standardised adjusted Group × Wave interaction effects for the three focal outcomesBlue circles represent point estimates, horizontal lines indicate 95% confidence intervals, and the vertical reference line denotes zero interaction effect. Positive estimates for MVPA and perceived physical literacy, and negative estimates for sitting time, favour the teamwork-oriented condition.

MVPA and sitting time showed different survey-wave patterns across conditions (Table 1). In adjusted models, the differential change was 67.15 min/week for MVPA (95% CI 2.23 to 132.07; *p* = 0.043; *q* = 0.058) and −87.08 min/day for sitting time (95% CI −159.28 to −14.88; *p* = 0.018; *q* = 0.054). The corresponding standardised Group × Wave interaction effects were 0.38 (95% CI 0.01 to 0.75) and −0.51 (95% CI −0.94 to −0.09), respectively (Table 2; Figure 2). Both estimates favoured the teamwork-oriented condition, but neither remained below the conventional *q* < 0.05 threshold after BH-FDR adjustment.

### 3.3. Survey-Wave Difference in Perceived Physical Literacy

The Group × Wave estimate for the PPLI total score is shown in Table 2. Mean PPLI scores were 30.63 ± 5.31 and 33.47 ± 5.29 at baseline and end-of-course in the teamwork-oriented condition, and 32.03 ± 4.76 and 32.67 ± 5.41, respectively, in the comparison condition. The adjusted differential change was 2.11 points (95% CI −0.07 to 4.29, *p* = 0.058), with a standardised effect of 0.42 (95% CI −0.01 to 0.85) and a BH-FDR-adjusted *q* value of 0.058. The estimate favoured the teamwork-oriented condition but remained imprecise (*q* = 0.058). None of the three PPLI subscales met *q* < 0.05 after BH-FDR correction within the secondary outcome family (Table S6).

### 3.4. Exploratory Results for Academic Burnout, Psychological Distress, and Sleep

No clear Group × Wave differences were detected for academic burnout, psychological distress, or sleep quality. None of the three MBI-SS dimensions, three DASS-21 dimensions, or PSQI total score met the BH-FDR-adjusted significance threshold (all *q* ≥ 0.806; Table S7).

### 3.5. Sensitivity Analyses

Sensitivity analyses showed consistent directions for the MVPA and sitting-time estimates across alternative specifications (Table S8). For MVPA, estimates were similar across covariate models, and both median regression (70.00 min/week, 95% CI 23.64 to 116.36; *p* = 0.003) and log-transformed analysis were directionally consistent. Sitting-time estimates were also stable across covariate and log-transformed models. The PPLI estimate showed greater sensitivity to covariate adjustment.

## 4. Discussion

### 4.1. Main Findings

Differences between the two course contexts were most evident for physical activity and sitting time. MVPA and sitting time showed favourable differential estimates that were directionally consistent across sensitivity analyses, whereas evidence for perceived physical literacy was less precise and no clear differential changes were detected for psychological or sleep outcomes. None of the three focal outcomes met *q* < 0.05 after BH-FDR correction; the findings should therefore be interpreted as favourable estimated patterns rather than confirmatory evidence of independent course effects. Given the complete confounding between instructor and course condition, the study identified patterns of differential change between two specific instructor–course contexts.

### 4.2. Potential Pathways Linking Teamwork-Oriented and Game-Enhanced Instruction to Physical Activity Behaviour

Teamwork-oriented organisation may plausibly influence activity behaviour through changes in participation structure. The positive interdependence, individual accountability, and promotive interaction emphasised in cooperative learning can enable students to maintain identifiable action responsibilities within shared tasks, thereby increasing opportunities for active participation and reducing low-participation states (Bores-García et al., 2021). In physical activity promotion research, peer support has shown a consistent association with physical activity levels among university students, and peer-based interventions have also demonstrated potential to promote exercise participation (Martin Ginis et al., 2013; Wang et al., 2024). Participation opportunities, accountability, social interaction, and game-related features are all plausible explanations, but the present design cannot distinguish their relative contributions.

Shared goals, immediate feedback, and visible task progress may further sustain engagement during exercise tasks. Meta-analytic evidence on gamified physical activity interventions shows small-to-moderate average effects but substantial heterogeneity across formats and settings (Mazéas et al., 2022). The contribution of game elements cannot be separated from the broader organisational features of the course. These mechanisms were not measured directly and should be tested in future mechanism-focused studies.

Sedentary behaviour requires separate interpretation. Increased physical activity and reduced sedentary behaviour do not represent the same behavioural process. Team-based organisation could reduce waiting or non-participation during lessons, although this was not directly measured. Research in university classrooms has shown that active learning and movement breaks can redistribute seated classroom time and reduce sedentary exposure (Lynch et al., 2022). Systematic reviews of physical activity interventions among university students likewise suggest that reorganising environments and opportunities for activity is an important implementation pathway for promoting participation (Johannes et al., 2024). However, the IPAQ-SF captured sitting over the preceding 7 days and cannot distinguish in-class from out-of-class time. The present data therefore support an association with whole-week activity and sitting patterns, but not localisation of the difference to a specific lesson component.

### 4.3. Perceived Physical Literacy: Favourable Direction, Limited Precision, and Educational-Setting Effects

Perceived physical literacy showed a favourable direction, but its change was less stable than the pattern observed for physical activity behaviour. This difference is theoretically plausible. Physical activity can adjust rapidly after changes in course structure, participation opportunities, and task demands, whereas perceived physical literacy involves ongoing judgements of physical competence, exercise confidence, and the ability to adapt to different activity environments; its development depends on repeated successful experiences, confirmation of competence, and reinterpretation of exercise contexts (Cairney et al., 2019; Sum et al., 2016). The less precise PPLI estimate may reflect slower or more variable self-perceptual change, although the present design cannot establish temporal sequencing.

This interpretation is consistent with the overall characteristics of existing physical literacy intervention research. Systematic reviews and meta-analyses indicate that physical literacy can be improved through educational interventions, but effects vary substantially across construct dimensions, implementation settings, and intervention formats (Carl et al., 2022). Meta-regression further suggests substantial variation across educational settings, indicating that intervention effects may depend on context, duration, and implementation (Ng et al., 2025). This heterogeneity also cautions against treating physical literacy as a unitary, rapidly responsive outcome.

The distinction between physical literacy and perceived physical literacy must also be maintained. The PPLI measures students’ subjective appraisal of their physical abilities, motivation and confidence, and capacity to interact with the environment (Ma et al., 2020). It cannot substitute for assessment of actual motor skills, objective physical competence, or comprehensive physical literacy. Existing research supports a consistent association between physical literacy and physical activity, including recent evidence among university students (C.-C. Huang et al., 2026), while longitudinal evidence suggests that physical literacy may predict subsequent activity participation (Park et al., 2025), largely outside medical education. Linked longitudinal studies combining perceived physical literacy, objective competence, and device-measured activity are needed to establish whether these changes precede, accompany, or follow behavioural change.

### 4.4. Interpretation of Findings for Mental Health and Sleep

No clear between-condition differences in survey-wave patterns were detected for psychological distress, burnout, or sleep quality. Psychological distress, burnout, and sleep among medical students are jointly shaped by multiple factors, including academic workload, assessment pressure, daily schedules, and individual susceptibility, and their fluctuations are therefore difficult to explain by a single course factor (Almutairi et al., 2022; Jahrami et al., 2023). Physical activity and sitting may be more proximal to course participation than psychological or sleep outcomes, but the present study did not test this pathway directly.

This finding is also consistent with the heterogeneity of research on physical activity interventions. Randomised trial evidence that exercise improves subjective sleep quality is relatively consistent (Xie et al., 2021). Systematic reviews and meta-analyses in university students generally support favourable effects of exercise on anxiety, depression, and stress, but effect sizes vary substantially by intervention dose, exercise type, duration, and target population, and the quality of the existing evidence is uneven (K. Huang et al., 2024; Singh et al., 2023). These studies generally evaluate exercise exposure itself, whereas the present contrast concerned how a common physical education curriculum was organised. Any psychological or sleep effects may depend on additional exposures or mechanisms not captured in this study.

Sleep is further constrained by differences in measurement timescale. The PSQI reflects overall sleep experience over approximately the previous month, whereas the DASS-21 primarily reflects recent psychological state; the two measures differ in their sensitivity to end-of-semester academic pressure and daily rhythms. Research on sleep in medical students likewise shows that sleep quality is highly dependent on stage of training, scheduling, and environmental factors (Binjabr et al., 2023; Rao et al., 2020). Because end-of-course surveys were administered one week apart, temporal context may also have contributed to between-condition variation. Accordingly, the absence of clear interaction effects should be interpreted as no detectable differential change in these outcomes, not as evidence of equivalence or absence of effect.

### 4.5. Implications for University Physical Education and Medical Education

The practical value of this study lies in using an existing curricular platform. Embedding such organisational approaches within compulsory physical education avoids creating a separate programme and may offer a pragmatic delivery model in time-constrained medical curricula. Future evaluations should align outcomes with the intended intervention target and distinguish proximal behavioural outcomes from broader health outcomes. Teamwork in physical education may have educational relevance for medical students, but clinical or interprofessional teamwork was not measured and should not be inferred.

### 4.6. Limitations

Several limitations warrant consideration. First, one instructor delivered each condition, precluding separation of instructor and course-condition effects. HC3 inference also assumed working independence of survey responses and did not account for potentially overlapping respondents or shared-class dependence. Second, non-random allocation at a single institution limited causal inference and generalisability. Third, unlinked surveys precluded within-student analyses, while unequal end-of-course participation may have influenced survey-wave comparisons. Fourth, final surveys occurred in different semester weeks (16 and 17), immediately before condition-specific examinations. Unverified teaching-session counts and differences in assessment timing or other concurrent factors may have introduced calendar-time confounding, particularly given the IPAQ-SF’s 7-day recall period. Fifth, self-reported activity and sitting time were susceptible to recall and social-desirability bias. Session-level game exposure, attendance, fidelity, and extracurricular exercise records could not be linked to survey responses, precluding exposure–response analyses. Finally, the study was not prospectively registered, and outcome hierarchies and multiplicity families were defined using the available dataset. Findings should therefore be interpreted alongside confidence intervals, multiplicity adjustment, and sensitivity analyses.

### 4.7. Future Research

Future studies should replicate this comparison across multiple classes and instructors per condition, ideally using cluster randomization or prospective allocation that permits instructor and course effects to be separated. Linked longitudinal follow-up, device-based activity monitoring, and session-level fidelity and attendance data would strengthen estimation of within-student change and attribution. If perceived physical literacy is examined as a mechanism, its temporal pathway should be preregistered and tested in adequately powered longitudinal models that also include objective physical competence.

## 5. Conclusions

In this two-wave unlinked course evaluation among first-year medical students, MVPA and sitting time showed favourable differential estimates for the teamwork-oriented, game-enhanced condition relative to the comparison condition. PPLI showed a favourable but imprecise estimate, whereas no clear differential changes were detected for academic burnout, psychological distress, or sleep quality. Given the non-randomised design and complete confounding of instructor with study condition, these findings should be interpreted as differences between two specific instructor–course contexts rather than as causal effects of the instructional approach.

## Figures and Tables

**Table 1:** Sample composition and baseline characteristics across the four survey cells.

Indicator	Teamwork-Oriented Condition Baseline (*n* = 94)	Teamwork-Oriented Condition End-of-Course (*n* = 92)	Comparison Condition Baseline (*n* = 92)	Comparison Condition End-of-Course (*n* = 76)	Baseline SMD
Age, years	18.69 ± 0.67	18.90 ± 0.70	18.62 ± 0.63	18.88 ± 0.56	0.111
Female, *n* (%)	63 (67.0%)	63 (68.5%)	65 (70.7%)	55 (72.4%)	−0.078
College, *n* (%)					
Basic Medical Sciences	11 (11.7%)	12 (13.0%)	5 (5.4%)	3 (3.9%)	0.225
Laboratory Medicine	8 (8.5%)	9 (9.8%)	12 (13.0%)	9 (11.8%)	−0.147
Public Health	19 (20.2%)	19 (20.7%)	23 (25.0%)	16 (21.1%)	−0.115
Pharmacy	9 (9.6%)	7 (7.6%)	10 (10.9%)	12 (15.8%)	−0.043
Medical Informatics	6 (6.4%)	6 (6.5%)	4 (4.3%)	3 (3.9%)	0.090
Pediatrics	11 (11.7%)	10 (10.9%)	7 (7.6%)	6 (7.9%)	0.139
First Clinical College	8 (8.5%)	7 (7.6%)	5 (5.4%)	2 (2.6%)	0.121
Second Clinical College	6 (6.4%)	6 (6.5%)	10 (10.9%)	10 (13.2%)	−0.160
Stomatology	9 (9.6%)	9 (9.8%)	11 (12.0%)	10 (13.2%)	−0.077
Fifth Clinical College	7 (7.4%)	7 (7.6%)	5 (5.4%)	5 (6.6%)	0.082
MVPA, min/week	149.73 ± 178.33	175.48 ± 160.30	151.07 ± 175.80	108.09 ± 135.57	−0.008
Sitting time, min/day	327.07 ± 164.75	286.65 ± 147.11	341.43 ± 173.91	389.30 ± 175.06	−0.085
PPLI total score	30.63 ± 5.31	33.47 ± 5.29	32.03 ± 4.76	32.67 ± 5.41	−0.279
DASS-21 total score	8.80 ± 6.82	8.10 ± 7.16	10.76 ± 8.23	11.62 ± 8.47	−0.260
PSQI total score	4.18 ± 2.10	4.66 ± 2.35	4.30 ± 3.10	5.41 ± 3.26	−0.046

Note: Continuous variables are presented as mean ± SD. SMDs describe baseline differences between conditions; baseline *p*-values were not used to assess comparability. College is presented as *n* (%) for the 10 categories represented in the analytic sample; five questionnaire-listed categories with no observations were omitted. College-specific baseline SMDs were calculated for each category as a binary indicator, with a maximum absolute SMD of 0.225. Because surveys were anonymous and unlinked, end-of-course responses were not paired with baseline responses.

**Table 2:** Adjusted Group × Wave models for focal outcomes.

Outcome	*n*	Adjusted Interaction Effect (95% CI)	Standardised Effect (95% CI)	*p*	BH-FDR *q*
MVPA, min/week	351	67.15 (2.23, 132.07)	0.38 (0.01, 0.75)	0.043	0.058
Sitting time, min/day	343	−87.08 (−159.28, −14.88)	−0.51 (−0.94, −0.09)	0.018	0.054
PPLI total score (range 8–40)	354	2.11 (−0.07, 4.29)	0.42 (−0.01, 0.85)	0.058	0.058

Note: All models were adjusted for sex, age, and college and used HC3 heteroskedasticity-robust standard errors. The interaction effect represents the between-condition difference in survey-wave change from baseline to end-of-course. MVPA, sitting time, and PPLI total score jointly constituted the multiple-comparison family of three focal outcomes, and the Benjamini–Hochberg procedure was used to control the false discovery rate. *p* values are unadjusted two-sided *p* values, and *q* values are the results of BH-FDR correction across the three focal outcomes.

## Data Availability

The de-identified dataset and analysis code will be made available upon reasonable request, subject to institutional and ethical approval.

## Supplementary Materials

The following supporting information can be downloaded at: https://www.mdpi.com/article/10.3390/bs16101948/s1, Supplementary File S1 (Intervention Manual) provides the intervention and comparison protocols, shared curricular framework, course chronology, representative team-game activities, implementation and fidelity information, and the completed TIDieR checklist. It contains Table S1: Shared components and key contrast between study conditions; Table S2: Planned curriculum, team-game activities, and course chronology. The separate supplementary statistical tables file contains Table S3: Data cleaning, scoring, and outcome-specific analytic availability; Table S4: Internal consistency estimates by study condition and survey wave; Table S5: Distributional summaries for the skewed focal behavioural outcomes; Table S6: Secondary physical activity outcomes and PPLI subscale analyses; Table S7: Exploratory academic burnout, psychological distress, and sleep outcomes; Table S8: Sensitivity analyses for the focal outcomes.
