# Supplementary material for: Teamwork-Oriented Physical Education Course Organisation and Survey-Wave Differences in Physical Activity, Sitting Time, and Perceived Physical Literacy Among Medical Students: A Two-Wave Unlinked Parallel-Group Evaluation
Source: Behav Sci (Basel). 2026 Oct 20;16(10):1948. doi: 10.3390/bs16101948 (PMC13701997; doi:10.3390/bs16101948)
Supplement: Supplementary file 1 [file behavsci-16-01948-s001.zip › Supplementary File S1.pdf]

Supplementary File S1

S1.1. Purpose and intervention contrast

This supplementary file provides a reproducible description of the intervention and comparison conditions. Both groups followed the same university Health and Physical Fitness Promotion curriculum, including the same 18-week content sequence, broad teaching objectives, and assessment framework. The principal contrast concerned instructional organisation. In the intervention condition, students were organised into small teams during each lesson, completed a structured team-based game aligned with the scheduled lesson content, and continued to participate in the main instructional and practice components through small-group organisation and peer-assisted task completion. The comparison condition followed the conventional instructional format without the systematic team-game component or the same structured team-based organisation.

S1.2. Shared curricular framework

The second-semester course included sports injury prevention and first aid, long-distance running, jumping, strength training, sprinting, physical fitness test practice, Tai Chi, and swimming. The two conditions therefore differed primarily in instructional organisation rather than in the prescribed curricular content.

Table S1. Shared components and key contrast between study conditions

Component	Intervention group	Comparison group
Course	Health and Physical Fitness Promotion	Same
Duration	18 scheduled weeks: 16 teaching weeks (weeks 1–16) and 2 assessment weeks (weeks 17–18).	Same
Core content	Theory; running; jumping; strength; sprinting; fitness-test practice; Tai Chi; swimming	Same
Broad objectives	Physical fitness, movement skills, and participation	Same
Assessment framework	University-prescribed course assessment	Same
Team-based game component	Structured team games systematically implemented and aligned with the scheduled lesson content	No systematic team-game component
Instructional organisation	Students were organised into small teams for team games and subsequent instructional and practice activities	Conventional instructional organisation without systematic team-based grouping
Team-collaboration design	Collaboration, peer interaction, and shared task completion systematically embedded	Not systematically embedded
Key contrast	Team-based gamified and small-group instructional organisation within the shared curriculum	Conventional instructional organisation within the shared curriculum

S1.3. Standard delivery protocol for the intervention group

During the 16-week instructional period, each 80-min intervention lesson followed the sequence below. The team-game component was allocated 25–30 min per lesson and was not implemented during the final two assessment weeks.

- Class assembly and safety briefing.
- Formation of small teams and completion of structured team games aligned with the scheduled lesson content.
- Main curricular instruction delivered through small-group organisation.
- Team-based or peer-assisted practice, with students completing instructional tasks collaboratively.
- Cool-down and brief teacher summary.

The 25–30-min game block comprised shorter tasks or rounds, as illustrated in Section S1.5. Small-group organisation continued into subsequent instruction and practice. Activities followed defined rules, completion criteria, participation requirements, and safety procedures. Groups generally comprised 4–8 students unless pair work was required.

S1.4. Planned curriculum and course chronology

Table S2. Planned curriculum, team-game activities, and course chronology

**Panel A. Weekly curricular content and corresponding intervention-group activities**			
**Week**	**Main lesson content**	**Team-based game/task**	**Primary function**
1	Sports injury prevention and first aid	Small-group risk-recognition task	Introduce safety and activate participation
2	Long-distance running	Team pacing challenge	Shared pacing and collective completion
3	Jumping	Grid invasion jumping game	Lower-limb activation, reaction, cooperation
4	Strength training	Team circuit challenge	Muscle activation and peer support
5	Sprinting	Fun relay	Acceleration, transitions, teamwork
6	Fitness-test practice 1	Testing-station rehearsal	Procedure familiarity and role allocation
7	Fitness-test practice 2	Group timing and recording challenge	Coordination and orderly participation
8	Fitness-test practice 3	Multi-station group task	Engagement and test familiarity
9	Tai Chi 1	Mirror-lead task	Attention, imitation, synchrony
10	Tai Chi 2	Silent formation change	Non-verbal coordination
11	Tai Chi 3	Three-person peer-teaching chain	Observation and peer feedback
12	Tai Chi 4	Movement error-detection task	Technical attention and correction
13	Tai Chi 5	Synchronous group display warm-up	Group consistency and rhythm
14	Tai Chi 6 (module assessment)	Rhythm activation and formation recall	State preparation before assessment
15	Swimming 1	Buddy safety-pairing task	Partner responsibility and safety
16	Swimming 2	Dry-land imitation and signal task	Movement rehearsal and communication
17	Course assessment	-	-
18	Course assessment	-	-
Panel B. Course chronology and survey timing			
	**Period (2026)**	**Scheduled course phase**	**Survey and examination**
1	2–8 March (week 1)	Start of teaching	Baseline surveys in both conditions, 4 March
2	9 March–14 June (weeks 2–15)	Scheduled teaching	No study surveys
3	15–21 June (week 16)	Final scheduled teaching week	Teamwork-oriented final survey and examination, 17 June
4	22–28 June (week 17)	First assessment week	Comparison final survey and examination, 24 June
5	29 June–5 July (week 18)	Second assessment week	No further study surveys

Note. Panel A presents the planned curriculum and representative intervention activities. Each intervention lesson included a prescribed 25–30-min team-game block comprising shorter tasks or rounds (Section S1.5). Panel B presents the scheduled course chronology. Final surveys preceded the respective examinations, occurring in week 16 for the teamwork-oriented condition and week 17 for the comparison condition. Actual teaching-session counts before these surveys were unavailable.

S1.5. Representative standardized team-game cards

The eight activity cards illustrate representative tasks within the 25–30-min game block. The stated 6–12-min durations refer to individual tasks or rounds rather than the full game component and were not independently timed during delivery.

Activity 1. Team pacing challenge

Module: Long-distance running

Typical setup: 6–8 students/team; cones; 8–10 min

Procedure: Teams complete a preset running distance while attempting to minimize spacing and finishing-time differences among members. Performance is based on coordinated completion rather than maximal speed.

Safety: Dynamic warm-up is completed first; no pushing or forced pacing; students may reduce intensity or stop if uncomfortable.

Instructional function: Prepares pacing awareness, peer support, and shared task engagement.

Activity 2. Grid invasion jumping game

Module: Jumping

Typical setup: Two teams of 6–8; floor grid/markers; 8–12 min

Procedure: One student from each team advances through marked squares by jumping. When opponents meet, rock–paper–scissors determines who continues; the other student returns and is replaced by a teammate. Reaching the opponent end zone scores one point.

Safety: One-square-at-a-time progression; adequate spacing; collisions and uncontrolled maximal jumps are prohibited.

Instructional function: Links jumping activation with turn-taking and team cooperation.

Activity 3. Team circuit challenge

Module: Strength training

Typical setup: 6–8 students/team; mats/bands/markers; 8–10 min

Procedure: Teams rotate through 3–4 low- to moderate-intensity activation stations (e.g., squats, planks, push-up holds). Peers assist with counting and simple movement checks; the goal is whole-team completion.

Safety: Technique takes priority over speed; task difficulty is reduced when needed.

Instructional function: Combines muscle activation with peer encouragement and shared completion.

Activity 4. Fun relay

Module: Sprinting

Typical setup: 6–8 students/team; baton/cones; 8–12 min

Procedure: Students complete short shuttle or relay runs and transfer the baton to the next teammate. Simple turning or cone-navigation elements may be added. Team performance is based on valid total completion time.

Safety: Clearly marked running and exchange zones; adequate deceleration space; no head-on running routes.

Instructional function: Prepares acceleration and transitions while promoting coordinated participation.

Activity 5. Testing-station rehearsal

Module: Physical fitness test practice

Typical setup: 6–8 students/team; test forms/markers; 8–10 min

Procedure: Students rotate through simplified test stations and alternate roles as performer, timer/counter, recorder, and organizer. At least one complete rehearsal cycle is performed.

Safety: Testing procedures and station boundaries are explained before practice; congestion is avoided.

Instructional function: Improves procedural familiarity, role clarity, and orderly participation.

Activity 6. Mirror-lead task

Module: Tai Chi

Typical setup: 4–6 students/group; no special equipment; 8–10 min

Procedure: One student leads simple upper-limb, trunk, and stepping patterns while peers mirror the movements. The leader role rotates after each short sequence.

Safety: Movements remain controlled and within a comfortable range; sudden high-speed twisting is avoided.

Instructional function: Prepares observation, rhythm, attention, and movement synchrony.

Activity 7. Silent formation change

Module: Tai Chi

Typical setup: 6–8 students/group; floor markers optional; 8–10 min

Procedure: Without speaking, group members reorganize into teacher-specified formations using observation and non-verbal coordination. Difficulty can progress from lines to simple geometric formations.

Safety: Walking rather than running; sufficient interpersonal spacing is maintained.

Instructional function: Develops non-verbal coordination and collective synchrony.

Activity 8. Buddy safety-pairing task

Module: Swimming

Typical setup: Pairs; routine swimming equipment; 6–8 min

Procedure: Paired students complete equipment checks, simple dry-land rehearsal, and confirmation of safety signals before the main swimming lesson. Both partners must complete the check before progressing.

Safety: All pool and venue safety rules apply; the activity does not replace instructor supervision or formal water-safety procedures.

Instructional function: Integrates partner responsibility and safety preparation into lesson entry.

S1.6. Design principles

The team-game activities were designed around four principles: (1) alignment with the scheduled main lesson; (2) rapid entry into active participation; (3) peer interaction through shared goals, role rotation, and mutual support; and (4) manageable challenge with explicit safety constraints. The module was therefore intended as a structured change in lesson organisation rather than a separate curriculum.

S1.7. Transparency and fidelity statement

The intervention protocol was documented in the course syllabus and teaching materials. Although the 25‑30-min game block was prescribed, realised duration, attendance, and session-level fidelity were not independently quantified for analysis. Extracurricular exercise time was recorded through routine check-ins but was not linked to the anonymous surveys. Intervention exposure and dose‑response associations therefore could not be assessed. The course chronology reflects scheduled weeks rather than verified teaching-session counts.

S1.8. TIDieR checklist

The checklist below maps the intervention description to the 12 TIDieR reporting items. It distinguishes the intended intervention specification from implementation features that were not quantitatively recorded.

TIDieR item	Reporting in this study	Location / clarification
1. Brief name	Teamwork-oriented, game-enhanced physical education course organisation within the Health and Physical Fitness Promotion curriculum.	Supplementary File S1, Sections S1.1–S1.2; main manuscript, Section 2.3.
2. Why	The organisational design used structured team games, small-group instruction, peer interaction, shared goals, and collaborative task completion to modify the participation structure of routine physical education.	Sections S1.1, S1.6; theoretical interpretation is presented cautiously in the main Discussion because mechanisms were not measured directly.
3. What: materials	Week-specific activities used routine physical education materials such as cones, floor markers, mats or bands, batons, test forms, and standard swimming equipment as applicable.	Sections S1.4–S1.5.
4. What: procedures	Lessons comprised assembly/safety briefing, a structured team-based game, main curricular instruction, team-based or peer-assisted practice, cool-down, and teacher summary.	Section S1.3; representative activity procedures are in Section S1.5.
5. Who provided	One university physical education instructor delivered the teamwork-oriented condition and another delivered the comparison condition as part of routine teaching. Both instructors were members of the same university physical education teaching unit and taught according to the same syllabus, curricular framework, core content, learning objectives, and assessment requirements. Instructor and condition were therefore completely confounded.	Main manuscript, Sections 2.1, 2.3, and 4.6. No instructor-level replication was available.
6. How	Face-to-face compulsory physical education delivered in intact course sections. The intervention condition used small teams, generally 4–8 students unless pair work was required.	Sections S1.1 and S1.3.
7. Where	The course was delivered within the routine university physical education setting at Chongqing Medical University.	Main manuscript, Section 2.1. Venue-specific characteristics were not used as analytic variables.
8. When and how much	The 18-week course comprised 16 teaching weeks and two assessment weeks. Each 80-min intervention lesson included a prescribed 25‑30-min team-game block, comprising shorter tasks or rounds. Realised game duration was not independently timed.	Sections S1.2‑S1.4 and Table S2. Actual teaching-session counts before the final surveys were unavailable.
9. Tailoring	Core curriculum content was shared across conditions. Within intervention activities, task difficulty could be reduced when needed, and week-specific variants followed the same delivery principles and safety constraints.	Sections S1.3 and S1.5.
10. Modifications	No prospectively documented intervention modifications were available as a separate quantitative record.	The supplement describes the intended standardised design and should not be interpreted as a session-by-session implementation log.
11. How well: planned	The intended protocol specified the weekly activity matrix, lesson sequence, group organisation, participation principles, completion criteria, and safety requirements.	Sections S1.3–S1.6 and Tables S1–S2.
12. How well: actual	Session-level game duration, attendance, fidelity, and in-class activity were not independently quantified for analysis. Extracurricular exercise check-in records were not linked to survey responses.	Section S1.7; main manuscript, Sections 2.3 and 4.6.
