# Supplementary material for: Teamwork-Oriented Physical Education Course Organisation and Survey-Wave Differences in Physical Activity, Sitting Time, and Perceived Physical Literacy Among Medical Students: A Two-Wave Unlinked Parallel-Group Evaluation
Source: Behav Sci (Basel). 2026 Oct 20;16(10):1948. doi: 10.3390/bs16101948 (PMC13701997; doi:10.3390/bs16101948)
Supplement: Supplementary file 1 [file behavsci-16-01948-s001.zip › Supplementary Tables S3-S8.pdf]

Supplementary Materials

Tables S1 and S2 are contained in Supplementary File S1 (the intervention manual). The statistical supplementary tables therefore continue with Table S3.

Table S3. Data cleaning, scoring, and outcome-specific analytic availability

Panel A. Data-cleaning, scoring, and analytic rules

Stage	Rule	Analytic impact	Rationale
Participation consent	Exclude responses that did not meet the consent criterion.	1 teamwork-oriented end-of-course response and 1 comparison baseline response excluded.	Ethics and informed-consent requirement.
Unlinked anonymous design	Do not infer individual matches across waves.	Cross-wave within-person correlation could not be identified or modelled; analyses therefore used a working-independence specification at the survey-response level.	Avoids unsupported individual matching without assuming that true within-person correlation was zero.
Platform option coding	Convert exported option indices for IPAQ day counts and MBI-SS, DASS-21, and PSQI frequency items to the questionnaire response scales.	All relevant items.	The survey platform exported response-option indices rather than scored values.
IPAQ-SF	Activity bouts <10 min were not counted; activity at a given intensity was truncated at 180 min/day; MET coefficients were 8.0, 4.0, and 3.3 for vigorous, moderate, and walking activity, respectively.	MVPA model n = 351; sitting-time model n = 343.	Pre-specified data cleaning to limit implausible extreme self-report values and follow common IPAQ processing conventions.
PSQI	When sleep duration or sleep-efficiency inputs could not be reconstructed unambiguously, only the PSQI total score was set to missing.	PSQI outcome model n = 351; seven-component reliability n = 347.	Outcome-specific missing-data handling retained other valid questionnaire outcomes.
Outcome-specific analytic availability	The online questionnaire required completion of all mandatory items before submission; therefore, no item-level nonresponse occurred among submitted questionnaires. Outcome-specific analyses used all records for which the corresponding outcome could be validly derived; no imputation was performed.	Model-specific n values were 351 for MVPA, 343 for sitting time, 351 for PSQI, and 354 for PPLI, MBI-SS, and DASS-21.	Differences in analytic n therefore reflect outcome-specific scoring or derivation availability rather than incomplete questionnaire submission.
Survey participation	Use the number of enrolled students in each condition as the denominator for wave-specific response rates.	Teamwork-oriented: 94/95 (98.9%) at baseline and 93/95 (97.9%) at end-of-course; comparison: 93/94 (98.9%) and 76/94 (80.9%), respectively.	Reports wave-specific participation without interpreting end-of-course nonresponse as individual attrition in the unlinked design.

Panel B. Outcome-specific analytic availability by study condition and survey wave

Outcome	Teamwork-oriented baseline (n = 94)	Teamwork-oriented end-of-course (n = 92)	Comparison baseline (n = 92)	Comparison end-of-course (n = 76)	Total valid n
IPAQ-SF activity-derived outcomes†	94 / 0 (0.0%)	92 / 0 (0.0%)	89 / 3 (3.3%)	76 / 0 (0.0%)	351
Sitting time	90 / 4 (4.3%)	88 / 4 (4.3%)	91 / 1 (1.1%)	74 / 2 (2.6%)	343
PSQI total score	93 / 1 (1.1%)	90 / 2 (2.2%)	92 / 0 (0.0%)	76 / 0 (0.0%)	351
PPLI total score and subscales	94 / 0 (0.0%)	92 / 0 (0.0%)	92 / 0 (0.0%)	76 / 0 (0.0%)	354
MBI-SS dimensions	94 / 0 (0.0%)	92 / 0 (0.0%)	92 / 0 (0.0%)	76 / 0 (0.0%)	354

DASS-21 dimensions and total score	94 / 0 (0.0%)	92 / 0 (0.0%)	92 / 0 (0.0%)	76 / 0 (0.0%)	354

Note. In Panel B, entries are valid n / unavailable n (% of survey-cell responses). Unavailable values reflect outcome-specific scoring or derivation constraints rather than incomplete questionnaire submission; no imputation was performed. †IPAQ-SF activity-derived outcomes comprise MVPA, total physical activity, vigorous-intensity activity, moderate-intensity activity, and walking, which shared the same analytic availability. IPAQ-SF = International Physical Activity Questionnaire–Short Form; MBI-SS = Maslach Burnout Inventory–Student Survey; DASS-21 = Depression Anxiety Stress Scales-21; PSQI = Pittsburgh Sleep Quality Index; PPLI = Perceived Physical Literacy Instrument; MVPA = moderate-to-vigorous physical activity; MET = metabolic equivalent of task.

Table S4. Internal consistency estimates by study condition and survey wave

Scale/subscale	Teamwork-oriented baseline	Teamwork-oriented end-of-course	Comparison baseline	Comparison end-of-course
PPLI total	94; α=.884, ω=.886	92; α=.929, ω=.933	92; α=.890, ω=.892	76; α=.944, ω=.945
PPLI confidence/physical competence	94; α=.842, ω=.846	92; α=.916, ω=.920	92; α=.798, ω=.806	76; α=.923, ω=.926
PPLI motivation	94; α=.755, ω=.773	92; α=.812, ω=.824	92; α=.780, ω=.806	76; α=.892, ω=.899
PPLI interaction with environment	94; SB=.920	92; SB=.896	92; SB=.799	76; SB=.950
MBI-SS exhaustion	94; α=.888, ω=.890	92; α=.925, ω=.926	92; α=.882, ω=.884	76; α=.927, ω=.927
MBI-SS cynicism	94; α=.822, ω=.802	92; α=.858, ω=.843	92; α=.925, ω=.919	76; α=.906, ω=.902
MBI-SS academic efficacy	94; α=.884, ω=.889	92; α=.904, ω=.905	92; α=.891, ω=.894	76; α=.933, ω=.935
DASS-21 depression	94; α=.836, ω=.845	92; α=.835, ω=.840	92; α=.884, ω=.889	76; α=.929, ω=.932
DASS-21 anxiety	94; α=.788, ω=.796	92; α=.844, ω=.856	92; α=.839, ω=.847	76; α=.860, ω=.864
DASS-21 stress	94; α=.797, ω=.803	92; α=.865, ω=.867	92; α=.874, ω=.884	76; α=.897, ω=.902
DASS-21 total	94; α=.897, ω=.898	92; α=.923, ω=.927	92; α=.930, ω=.934	76; α=.934, ω=.934
PSQI seven components	92; α=.570	90; α=.493	91; α=.785	74; α=.749

Note. Entries are n and reliability coefficients. α = Cronbach's alpha; ω = McDonald's omega; SB = Spearman–Brown coefficient. The PPLI interaction-with-environment subscale contains two items and therefore uses the Spearman–Brown coefficient. For PSQI, pooled seven-component reliability was n = 347, α = .690. IPAQ-SF was not evaluated with internal-consistency coefficients because it comprises distinct behavioral domains rather than interchangeable items.

Table S5. Distributional summaries for the skewed focal behavioural outcomes

Outcome	Study cell	Median [Q1, Q3]
MVPA, min/week	Teamwork-oriented baseline	110.0 [60.0, 180.0]
MVPA, min/week	Teamwork-oriented end-of-course	125.0 [60.0, 240.0]
MVPA, min/week	Comparison baseline	100.0 [60.0, 165.0]
MVPA, min/week	Comparison end-of-course	72.5 [20.0, 145.0]
Sitting time, min/day	Teamwork-oriented baseline	300.0 [210.0, 360.0]
Sitting time, min/day	Teamwork-oriented end-of-course	300.0 [180.0, 360.0]
Sitting time, min/day	Comparison baseline	300.0 [200.0, 480.0]
Sitting time, min/day	Comparison end-of-course	360.0 [240.0, 480.0]

Note. MVPA = moderate-to-vigorous physical activity; Q1 and Q3 = first and third quartiles. These distributional summaries complement the mean ± SD values reported in the main manuscript.

Table S6. Secondary physical activity outcomes and PPLI subscale analyses

Outcome	Model scale	N	Group × wave estimate (95% CI)	p	BH-FDR q
Total physical activity, MET-min/week	log(1 + x)	351	0.496 (−0.010, 1.003)	.055	.096
Vigorous physical activity, min/week	log(1 + x)	351	0.765 (−0.059, 1.589)	.069	.096
Moderate physical activity, min/week	log(1 + x)	351	0.822 (0.073, 1.572)	.032	.095
Walking, min/week	log(1 + x)	351	−0.033 (−0.561, 0.496)	.904	.904
PPLI confidence/physical competence	Raw score	354	0.942 (0.040, 1.844)	.041	.095
PPLI motivation	Raw score	354	0.379 (−0.454, 1.211)	.373	.435
PPLI interaction with environment	Raw score	354	0.789 (0.047, 1.531)	.037	.095

Note. Models adjusted for sex, age, and college using HC3 robust standard errors. Confidence intervals and p values were conditional on working independence of survey responses, without adjustment for respondent overlap or class clustering. The seven secondary outcomes formed one family for Benjamini–Hochberg FDR correction; none met $q < 0.05$. PPLI = Perceived Physical Literacy Instrument.

Table S7. Exploratory academic burnout, psychological distress, and sleep outcomes

Outcome	N	Group × wave estimate (95% CI)	p	BH-FDR q
MBI-SS exhaustion	354	−0.143 (−0.596, 0.310)	.536	.806
MBI-SS cynicism	354	−0.019 (−0.460, 0.421)	.932	.932
MBI-SS academic efficacy	354	−0.143 (−0.644, 0.358)	.575	.806
DASS-21 depression	354	−0.804 (−2.120, 0.513)	.232	.806
DASS-21 anxiety	354	−0.136 (−1.345, 1.073)	.825	.932
DASS-21 stress	354	−0.411 (−1.734, 0.912)	.542	.806
PSQI total	351	−0.580 (−1.790, 0.630)	.347	.806

Note. Models adjusted for sex, age, and college using HC3 robust standard errors. Confidence intervals and p values assumed working independence of survey responses, without adjustment for respondent overlap or class clustering. The seven exploratory outcomes formed one family for Benjamini–Hochberg FDR correction. Higher MBI-SS academic efficacy scores indicate more favourable outcomes, whereas higher exhaustion, cynicism, DASS-21, and PSQI scores indicate less favourable outcomes.

Table S8. Sensitivity analyses for the focal outcomes

Analysis	n	Estimate (95% CI)	p
MVPA: unadjusted	351	68.720 (0.246, 137.193)	.049
MVPA: sex + age	351	67.601 (1.310, 133.892)	.046
MVPA: sex + age + college	351	67.148 (2.230, 132.066)	.043
Sitting time: unadjusted	343	−88.288 (−159.215, −17.361)	.015
Sitting time: sex + age	343	−86.793 (−157.725, −15.862)	.016
Sitting time: sex + age + college	343	−87.080 (−159.276, −14.883)	.018
PPLI total: unadjusted	354	2.201 (0.011, 4.391)	.049
PPLI total: sex + age	354	2.130 (−0.034, 4.294)	.054
PPLI total: sex + age + college	354	2.110 (−0.073, 4.293)	.058
MVPA: adjusted median regression	351	70.000 (23.643, 116.357)	.003
MVPA: adjusted log(1 + x) OLS-HC3	351	0.965 (0.273, 1.658)	.006
Sitting time: adjusted log(1 + x) OLS-HC3	343	−0.343 (−0.655, −0.032)	.031

Note. These analyses assess robustness to covariate adjustment and alternative distributional specifications. Unless otherwise stated, adjusted models included sex, age, and college. Estimates for raw-scale MVPA and sitting time are expressed in min/week and min/day, respectively; PPLI estimates are in raw-score points, and log(1 + x) estimates are on the transformed scale. BH-FDR-adjusted q values for the three focal outcomes are reported in Table 2 of the main manuscript.
